# The Mediating Effects of Stigma on Depressive Symptoms in Patients With Tuberculosis: A Structural Equation Modeling Approach

**DOI:** 10.3389/fpsyt.2018.00618

**Published:** 2018-11-26

**Authors:** Lei Qiu, Qin Yang, Yeqing Tong, Zuxun Lu, Yanhong Gong, Xiaoxv Yin

**Affiliations:** ^1^School of Public Health, Tongji Medical College, Huazhong University of Science and Technology, Wuhan, China; ^2^School of management, Hainan Medical University, Haikou, China; ^3^Center for Disease Control and Prevention of Hubei Province, Wuhan, China

**Keywords:** tuberculosis, stigma, depression, epidemiology, China

## Abstract

**Objectives:** To date, the complex interrelationships between family function, doctor-patient communication, knowledge about tuberculosis (TB), stigma, and depressive symptoms among patients with TB are insufficiently understood. We explored the interrelationships between family function, doctor-patient communication, knowledge about TB, TB-related stigma, and depressive symptoms and examined whether TB-related stigma played a mediating role.

**Methods:** A cross-sectional survey was conducted between October 1, 2013 and March 31, 2014 in Hubei province, central China. Data were collected from 1,309 patients with TB using a structured questionnaire that measured family function, doctor-patient communication, knowledge about TB, stigma, and depressive symptoms. Structural equation modeling was used to examine the interrelationships among the study variables based on the hypothesized model.

**Results:** The proposed model provided a good fit to the obtained data. There were indirect effects between family function, doctor-patient communication, knowledge about TB, and depressive symptoms through stigma (β = −0.048, *P* = 0.002; β = −0.028, *P* = 0.001; β = −0.021, *P* = 0.009, respectively). Stigma partially mediated the effect of family function and knowledge about TB on depressive symptoms and fully mediated the effect of doctor-patient communication on depressive symptoms.

**Conclusions:** This study elucidated the pathways linking family function, doctor-patient communication, and knowledge about TB to depressive symptoms and confirmed that the effect of those variables on depressive symptoms can be mediated by stigma. Those findings provide direction and information for depression interventions among patients with TB.

## Introduction

Tuberculosis (TB) remains a major global health problem (1, 2). TB affects not only physical health, but also the mental well-being of patients. For patients with TB, depression is a common mental disorder (3). The presence of depressive disorder leads to poor treatment adherence (4), drug resistance, and high rates of community transmission, ultimately increasing morbidity, and mortality (5). Therefore, it is necessary to identify the related risk factors of depression among patients with TB and accordingly devise effective interventions.

At present, studies have begun to explore the influencing factors of depression in patients with TB to take measures to reduce the risk of depression. However, the prevalence of depressive symptoms in patients with TB is still high, as reported in India (39.5%) (6), Ethiopia (43.4%) (7), Pakistan (46.3%) (8), and Cameroon (61.1%) (9). Much of the existing TB programs were focused on outcomes of mortality and microbiological cure (10), while mental health-related outcomes were ignored, which may lead to the high prevalence of depression in patients with TB. In addition, inadequate research on the influencing factors of depression may be another key reason. Previous studies have reported that some personal and social factors were associated with depression in patients with TB, such as age, sex, lack of TB knowledge, insufficient social support, and TB-related stigma (7, 9). However, it is unclear whether these factors act independently or through complex mechanisms. Furthermore, previous studies exploring factors associated with depression mainly relied on analysis via logistic regression or linear regression analyses (9, 11). Although useful information was provided, these types of analyses considered the effects among the variables that may affect depression independently; However, it is possible that those variables interacted in a more complex way, which was not straightforwardly captured by the regression analysis. Therefore, the complexity of the interrelationships among them is less well-understood (12).The lack of research on those complex relationships makes it difficult to determine accurately what variables should be targeted in depression interventions (13).

An alternative method to tackle these issues is the use of structural equation modeling (SEM), a technique designed to decompose into direct and indirect effects of variables to understand mechanisms and pathways that may explain these relationships (14, 15). In addition, SEM analysis is generally more robust than regression analyses because measurement errors are considered (16). Consequently, we applied SEM to address the complex interrelationships between specific personal and social variables and depressive symptoms among patients with TB in China. Understanding complex pathways may increase the effectiveness of interventions to reduce depression risk among patients with TB, which may ultimately decrease the morbidity and mortality.

## Theoretical background

### Stigma and depressive symptoms

Goffman posited that stigma was a social process that reduced individuals “from a whole and usual person to a tainted, discounted one” (17). During this process, patients tend to conceal their symptoms and withdraw from interpersonal contact for fear of discrimination, thereby isolating themselves to avoid negative public attitudes. TB is usually associated with uncleanliness, and patients are often attached to a disease label and experience social stigma (18, 19). Previous studies on the impact of stigma among patients with TB suggested TB-related stigma may lead to diminished self-esteem and self-efficiency and has been found to be significantly associated with the development of mental health problems, especially suffering from depressive symptoms (20, 21). Moreover, a number of studies have confirmed that patients with TB are more likely to have depressive symptoms than the general population and a high level of stigma among patients with TB in different countries has been reported (7, 9, 20). However, few studies have investigated the status of stigma and their relationship with depression in patients with TB in China. Due to variation in socio-cultural elements, ethnicity, and region, factors associated with TB-related stigma may differ and it is necessary to focus on the stigma and depression of patients with TB in China.

### Personal and social factors (e.g., family function, doctor-patient communication, knowledge about TB, etc.) and depressive symptoms

The evidence for the relationship between social support and depressive symptoms has been well-substantiated by previous studies (7, 11). Indeed, the family is the main source of social support when the disease occurs, whether through tangible instrumental support, such as administering medication and preparing meals, or through emotional support (22). A-well functioning family suggests that the family could adapt to the crisis including the patients illness and role change and may be effective in lowering the risk of depression, while family dysfunction would suggest that the home environment might be stressful to the patient with TB. In addition, good doctor-patient communication is also a source of social support for patients. Previous studies has demonstrated that the levels of perceived social support received from healthcare workers are negatively associated with depressive symptoms (23). Jittimanee et al. found that patients with low TB knowledge were more likely to have severe TB disease and to have depressive symptoms (24).

### Personal and social factors (e.g., family function, doctor-patient communication, knowledge about Tb, etc.) and stigma

Various researchers have explored the relationship between personal and social factors (e.g., family function, doctor-patient communication, knowledge about TB, etc.) and stigma in patients with TB. The relationship between social support and TB-related stigma has been examined and low levels of social support was associated with high levels of TB-related stigma in patients with TB (25). Few studies, however, have been published to date on the relationship between family function and TB-related stigma. Only one study conducted in Pakistan showed that good family function can alleviate stigma in patients with TB (26). Stewart et al have reported doctor-patient communication may have negative correlation with stigma (27). Moreover, in our previous study, the relationship between patients' knowledge about TB and stigma in patients with TB has been confirmed. We found that knowledge about TB may have negative correlation with stigma using logistic regression (28).

## Model assignment and hypotheses

In summary, previous studies provided some evidence that TB-related stigma are influenced by personal and social variables (e.g., family function, doctor-patient communication, knowledge about TB, etc.) (28, 29). Previous studies also provided evidence that patients with greater TB-related stigma were more likely to have higher levels of depression (7). However, the complex interrelationships between these variables have not been explored.

**Figure 1:**
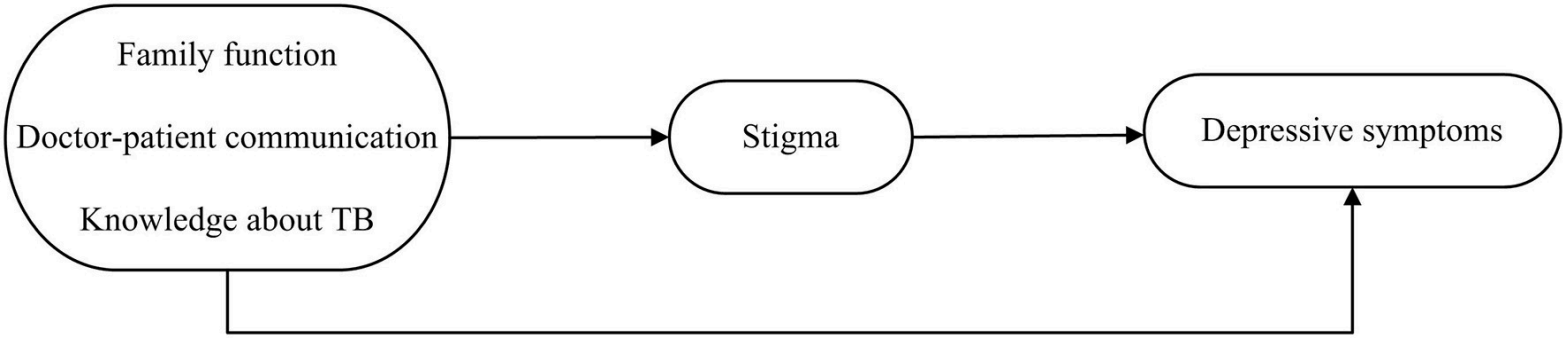
Hypothetical model of relationships between family function, doctor-patient communication, and knowledge about TB, stigma, and depression among patients with TB.

Based on the above review, a hypothetical model (Figure 1) was obtained. As illustrated in Figure 1, we proposed the following hypotheses: (Hypothesis 1) Family function, doctor-patient communication, and knowledge about TB will be negatively related to stigma. (Hypothesis 2) Stigma will be positively related to depressive symptoms. (Hypothesis 3) Stigma will mediate the relationship between family function, doctor-patient communication, knowledge about TB, and depressive symptoms.

## Methods

### Participants and sampling

Ethical approval was provided by the Human Research Ethics Committee, Huazhong University of Science and Technology, Wuhan, China. Each participant was voluntary and provided written informed consent prior to participating in the study.

A cross-sectional study was carried out between October 1, 2013 and March 31, 2014 in Hubei province, central China. Multistage stratified sampling was performed. Counties across Hubei province were divided into three categories based on their economic development status (i.e., upper, middle, and lower), and a county was randomly selected from each category. Thereafter, patients with TB who attended TB clinics at the study sites in the selected counties were recruited as participants. The inclusion criterion was a diagnosis of active TB, based on national TB program guidelines, and the exclusion criterion was the presence of psychosis. Some elderly people with TB have more health problems, which makes it very difficult for them to complete the self-assessment questionnaire, resulting in a valid response rate of 84.7%. Thus, 1,430 patients with TB completed a structured, self-administered questionnaire anonymously. Of the collected 1,430 questionnaires, 88 were removed due to a large number of missing data, and eventually 1,342 TB patients were included in the analysis.

### Measuring instruments

The questionnaire consisted of six sections: Demographic Characteristics, Family Function, Doctor-patient Communication, Knowledge about TB, TB-Related Stigma, Depressive Symptoms. The demographic characteristics included age, sex, educational level, and history of prior anti-TB treatment.

Patients' family function was measured using the Family APGAR Index developed by Smilkstein in 1978 (30). It was designed to evaluate the satisfaction with social support received from their family members based on five components: Adaptability, Partnership, Growth, Affection, and Resolve. Each item is scored using a 3-point Likert scale ranging from “scarcely” (= 0) to “often” (= 3). The item scores are summed to provide a total score (range: 0–10) and a higher score indicates better family function. The Family APGAR Index has been widely used in China, with good reliability and validity (31). In the present study, the Family APGAR Index showed high internal consistency (Cronbach's α = 0.86).

Doctor-patient communication was measured by the following four questions: (1) satisfaction with the doctors' service attitude; (2) extent to which the doctor introduces the illness; (3) extent to which the doctor explains the details of taking anti-TB agents; and (4) extent to which the doctor explains adverse drug reactions to anti-TB agents. Patients were asked to rate their answers in terms of satisfaction or detail scale ranging from 1 to 3 for the four questions listed above. The total score ranged from 4 to 12, and a higher score means better doctor-patient communication.

Patients' knowledge about TB was measured by six multiple-choice questions which were mainly from the questionnaire used in the National TB Epidemiological Survey of China (32): (1) the cause of TB, (2) the route of TB transmission, (3) whether TB can be cured, (4) duration of TB standard treatment regimens, (5) common clinical symptoms of TB, and (6) unhealthy behaviors that make TB susceptible to infection.

In the six questions, the first four questions have a single correct answer, and the patient scores 1 point for each correct answer; There were four correct answers to the remaining two questions, and the patient receives 0.5 point for each correct choice. If the answer is incorrect or “I do not know,” then the question score is 0 points. Thus, the total score of TB knowledge ranges from 0 to 8 points. The higher the TB knowledge score reflects that the patient has more knowledge of TB.

TB-related stigma was measured using the TB-Related Stigma Scale, which was developed by our research group according to the standard method for the development of new scales and has shown good validity and internal consistency (Cronbach's α = 0.88). Detailed information on the development and evaluation of this scale was available in our previous study (33). The scale consists of nine items divided between three subscales (Negative Experience, Emotional Reactions, and Coping Style). Responses for the items are provided using a 4-point Likert scale ranging from “strongly disagree” (= 0) to “strongly agree” (= 3). The item scores are summed to provide a total score (range: 0–27), and higher scores indicate greater stigma.

Depressive symptoms were measured using the Center for Epidemiologic Studies Depression (CES-D) Scale, developed to identify individuals who are at risk for depression (34). The scale consists of 20 items divided between four subscales: Depressive Mood, Somatic Symptoms, Interpersonal Relationships, and Positive Affect. Responses for the items are provided using a 4-point Likert scale ranging from “not at all” (= 0) to “almost daily” (= 3). The item scores are summed to provide a total score (range: 0–60). The CES-D has been widely used in china (35) and in the current study the scale demonstrated high internal consistency (Cronbach's α = 0.86).

### Data analyses

Descriptive statistics comprised percentages, means, and standard deviations (SD). Student's *t*-test and analyses of variance were conducted to compare depressive symptoms scores between groups. Bivariate correlations were assessed using Pearson's correlation. The above analyses were conducted using SAS 9.4 software (SAS Institute Inc., Cary, NC). All differences were assessed using two-tailed tests, and the significance level was set at *P* < 0.05.

SEM was conducted using AMOS 17.0 software to assess the interrelationship between variables associated with depressive symptoms in patients with TB. In addition, the significance of direct and indirect effects was examined using a bias-corrected bootstrap 95% confidence interval (CI). The following fit indices were used to assess the overall model fit: root mean square error of approximation (RMSEA), comparative fit index (CFI), and Tucker-Lewis index (TLI). CFI and TLI values above 0.90 and RMSEA values below 0.08 indicated acceptable fit; CFI and TLI values above 0.95 and RMSEA values below 0.05 indicated good fit (36).

## Results

### Participants' characteristics

Participants' characteristics and their association with depressive symptoms are presented in Table 1. Participants mean age was 47.72 (SD = 17.06) years and most were aged 45 years or older. The mean score of depressive symptoms was 15.84 (SD = 8.26) and 48.00% of the participants had depressive symptoms. Being older, female, having low education, and a history of prior anti-TB treatment were associated with higher depressive symptoms scores.

**Table 1:** Participants' characteristics and their associations with depressive symptom (*n* = 1342).

**Variables**	**All subject**		**Depressive symptoms**		***P*-value**
	***n***	**%**	**mean**	**SD**
Age(Missing = 14)					0.000
11~29	283	21.31	13.79	7.85
30~44	231	17.39	16.42	8.20
45~59	440	33.13	16.04	8.00
60+	374	28.16	16.84	8.52
Sex					0.000
Male	905	67.44	15.24	8.12
Female	437	32.56	17.08	8.43
Education					0.000
Primary or less	574	42.77	17.50	8.50
Secondary	540	40.24	15.30	7.98
High school or higher (above)	228	16.99	13.00	7.36
History of prior anti-TB treatment					0.025
No	1174	87.48	15.65	8.16
Yes	168	12.52	17.20	8.88

*SD, standard deviations; TB, tuberculosis*.

### Correlations of the variables, test of normality, and multicollinearity

Table 2 shows the means, SDs, and correlations among study variables. There were significant correlations between all study variables in the bivariate analysis. The correlation between family function and depressive symptoms was high compared to all the other combinations of correlations. Furthermore, skewness and kurtosis were used to examine the assumption of normality. The skewness of all study variables was within 1.90, and the absolute value of kurtosis was within 3.00; therefore, the results satisfy the assumption of a normal distribution (37). In addition, diagnostic checks for multicollinearity were conducted through variance inflation factors. In this study, the variance inflation factors ranged from 1.07 to 1.14, suggesting that there was no problem with multicollinearity (38).

**Table 2:** Means, standard deviations, and correlations among study variables.

**Variables**	**Mean(SD)**	**Family function**	**Doctor-patient communication**	**Knowledge about TB**	**Stigma**
Family function	7.58 (2.39)			
Doctor-patient communication	11.34 (1.24)	0.22^**^		
Knowledge about TB	4.84 (2.02)	0.25^**^	0.16^**^	
Stigma	9.27 (4.25)	−0.23^**^	−0.15^**^	−0.16^**^
Depressive symptoms	15.84 (8.26)	−0.45^**^	−0.21^**^	−0.34^**^	0.28^**^

TB, tuberculosis; SD, standard deviations; All correlations were significant (p < 0.01);

** *p < 0.01*.

### Effect analysis of hypothesized model

#### The first model without mediator

**Figure 2:**
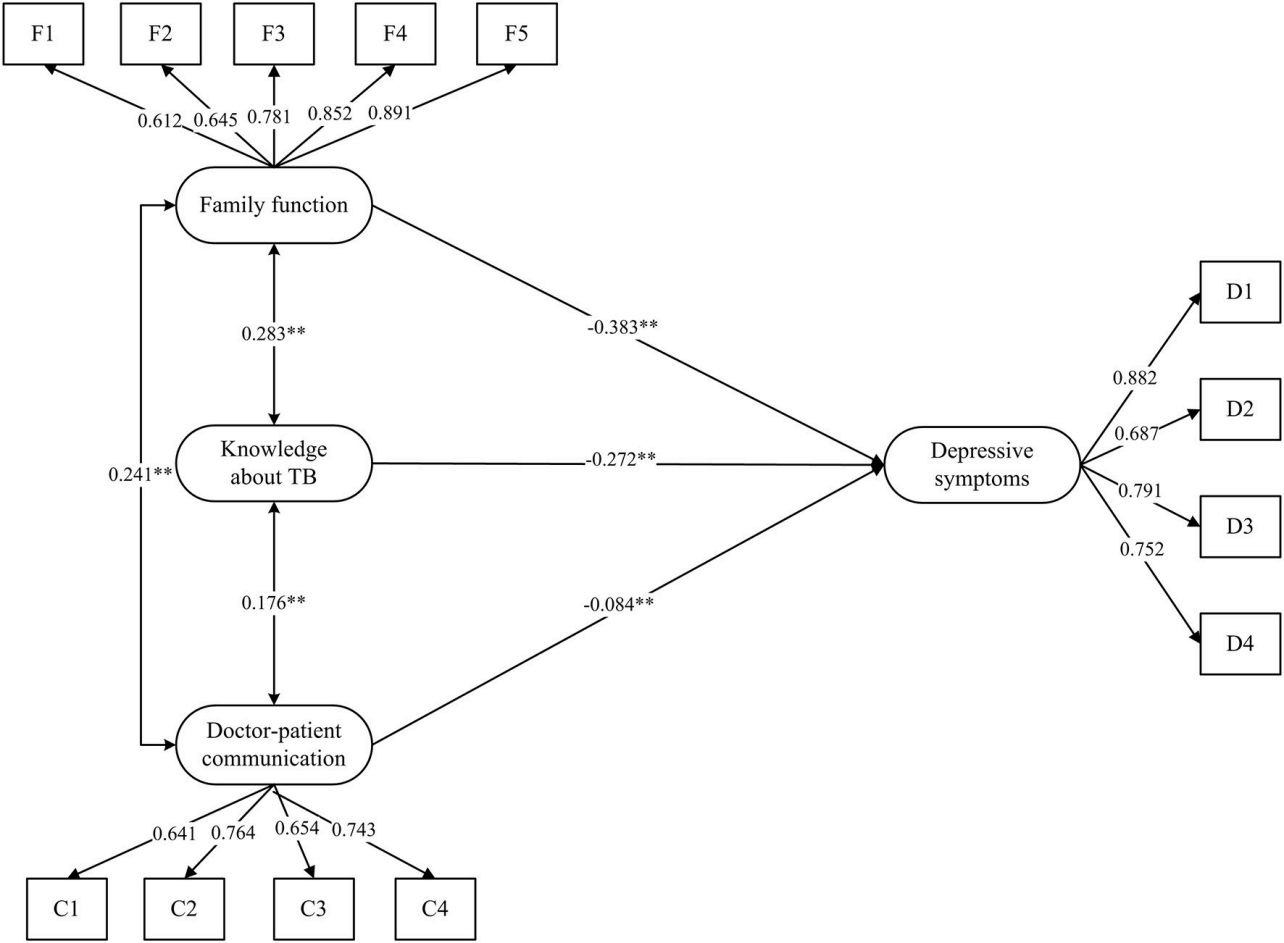
The first model without mediator. Estimates were obtained using full information maximum likelihood and all the coefficients in the figure were standardized. Five dimensions of the Family APGAR Index labeled F1,F2, F3, F4, and F5. Four questions of the doctor-patient communication Scale labeled C1, C2, C3, and C4. Four subscales of the Center for Epidemiologic Studies Depression (CES-D) Scale labeled D1, D2, D3, and D4. ^**^p < 0.01.

In the first model, the direct effects of the three variables (family function, doctor-patient communication, and knowledge about TB) on the dependent variable (depressive symptoms) were tested without a mediator using SEM. Figure 2 showed the first model with standardized path coefficients. We found that family function, doctor-patient communication, and knowledge about TB were negatively associated with depressive symptoms (standardized path coefficients were −0.38, −0.27, −0.08, respectively). The overall model fit indices of the first model were RMSEA = 0.039, CFI = 0.981, NFI = 0.972, TLI = 0.974, which all indicate that the model fit well.

#### The final model

**Figure 3:**
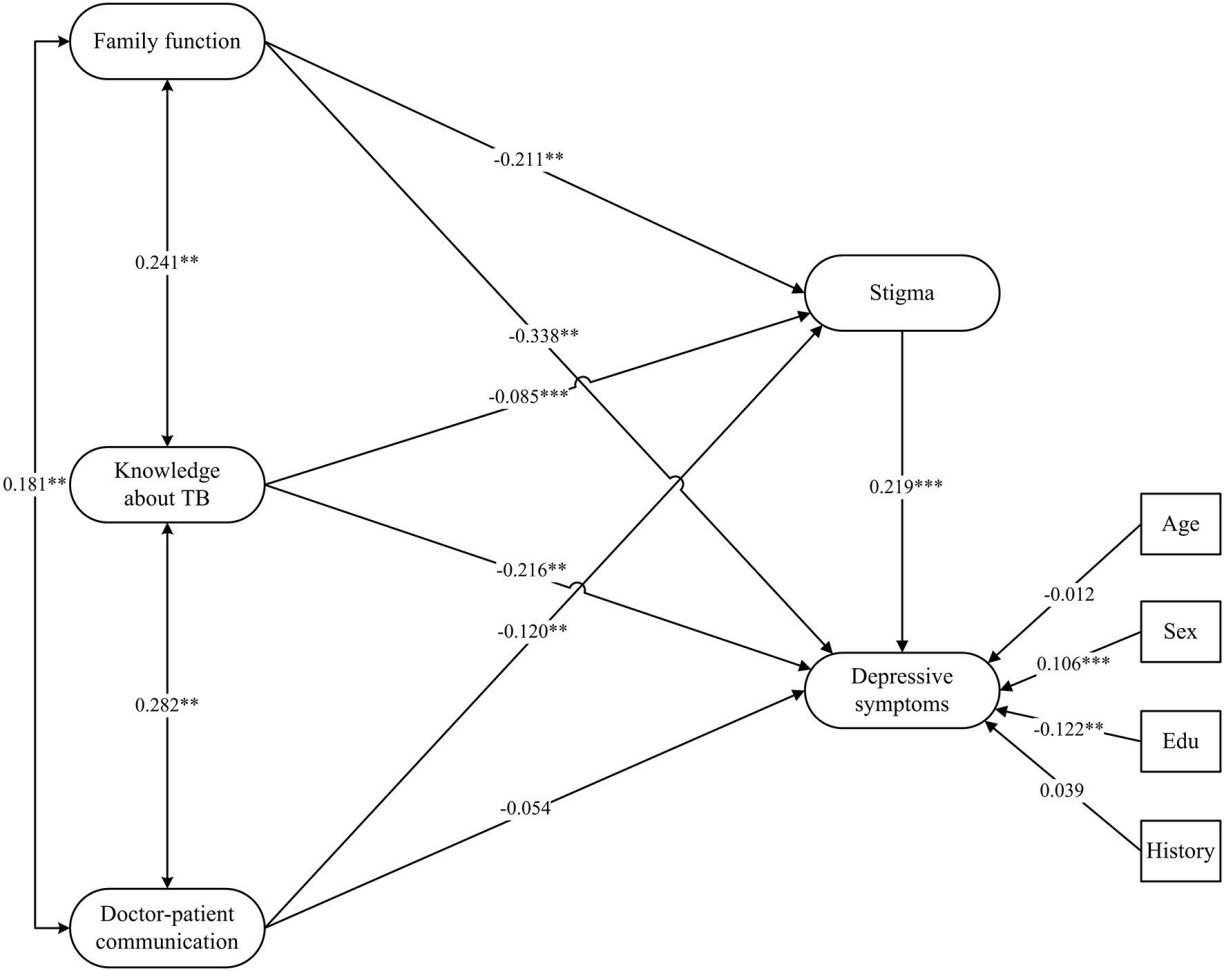
The final model. Estimates were obtained using full information maximum likelihood and all the coefficients in the figure were standardized; Age, Sex, Education, and History of prior anti-TB acted as covariates; Edu, Education; History, History of prior anti-TB (History); ^**^p < 0.01; ^***^p < 0.001.

Figure 3 shows the final model with standardized path coefficients. The significance of all the effects were examined using a 95% bootstrapped confidence interval estimate. Age, sex, education, and history of prior anti-TB acted as covariates. The summary of the results are provided in Table 3. The overall model fit indices of the final model were RMSEA = 0.043, CFI = 0.956, NFI = 0.939, TLI = 0.946, which all indicate that the model fitted well.

**Table 3:** The results of hypothesized paths for the final model.

**Model paths**	**Standardized direct effects**	**95% CI**		**Standardized indirect effects**	**95% CI**	
Family function → Stigma	−0.211^**^	−0.276	−0.135	–	–	–
Doctor-patient communication → Stigma	−0.120^**^	−0.192	−0.051	–	–	–
Knowledge about TB → Stigma	−0.085^**^	−0.146	−0.019	–	–	–
Stigma → Depressive symptoms	0.219^**^	0.160	0.280	–	–	–
Family function → Depressive symptoms	−0.338^***^	−0.401	−0.271	−0.048^**^	−0.065	−0.029
Doctor-patient communication → Depressive symptoms	−0.054	−0.118	0.007	−0.028^**^	−0.045	−0.011
Knowledge about TB → Depressive symptoms	−0.216^**^	−0.273	−0.162	−0.021^**^	−0.034	−0.005

TB, tuberculosis; CI, confidence interval; All of the effects were examined using a bias-corrected bootstrap 95% confidence interval; All of the indirect effects were significant (p < 0.01);

** p < 0.01;

*** *p < 0.001*.

As can be seen in Table 3, the direct effect of family function, doctor-patient communication, and knowledge about TB on stigma were significant, which supported hypothesis 1. In addition, stigma exerted a direct effect on depressive symptoms, which supported hypothesis 2. The direct effect of family function and knowledge about TB on depressive symptoms were both significant; however, the direct effect of doctor-patient communication on depressive symptoms was non-significant. Further, the indirect effect of family function, doctor-patient communication, and knowledge about TB on depressive symptoms were significant; therefore, stigma partially mediated the effect of family function and knowledge about TB on depressive symptoms and fully mediated the effect of doctor-patient communication on depressive symptoms, which supported hypothesis 3.

## Discussion

To the best of our knowledge, this was the first study to test the mediating effects of stigma on depressive symptoms in patients with TB. We examined the interrelationships between family function, doctor-patient communication, knowledge about TB, stigma, and depressive symptoms. More importantly, we elucidated the pathways linking family function, doctor-patient communication, and knowledge about TB to depressive symptoms and confirmed that the effect of those variables on depressive symptoms can be mediated by stigma, which supported our hypotheses.

Previous studies indicated that family function and doctor-patient communication were the most critical sources of social support for patients (39). Our results showed that family function and doctor-patient communication can also have an indirect negative effect on depression symptoms through stigma. Good family function and doctor-patient communication can increase patient life satisfaction and social confidence. Patients with family dysfunction and poor doctor-patient communication are more susceptible to being isolated and estranged. Because of these negative treatments, patients have developed negative feelings such as shame, guilt, discrimination, etc., which may result in a sense of stigma (40). Moreover, prominent levels of stigma was associated with psychological stress disorder, which can increase their risk of psychological problems, such as depression symptoms (20).

Knowledge about TB was another influencing factor that had both direct and indirect effects on depressive symptoms. This indirect negative effect was also achieved through stigma. Inadequate disease-related knowledge often represented the absence of a correct understanding of TB, leading to impaired confidence in treatment and diminished self-efficacy (41). Patients with low self-efficacy also experienced greater stigma (42), which contributed to their elevated levels of depression.

The present study is notable in that it disentangled the pathways between family function, doctor-patient communication, knowledge about TB, and depressive symptoms. Understanding these factors and their interrelationships offer an opportunity to effectively intervene in depression among patients with TB and interventions can be tailored to these specific pathways. Specifically, interventions aimed at reducing depression among patients with TB should focus on improving patients' family function, doctor-patient communication, and knowledge about TB. More importantly, the role of stigma should be understood and addressed. Given that stigma was not only a negative psychological reaction (18), but also mediated the effect of other factors on the mental health of patients with TB, interventions should be combined stigma-reduction measures, which is essential for promoting both physical and psychological well-being and, ultimately, better health outcomes. Specific stigma-reduction measures include routine screening for stigma of patients with TB, giving patients access to education on the concept of stigma, and offering techniques to deal with it.

This study had several limitations. First, analysis of cross-sectional data limits our ability to establish causal relationships among study variables. In addition, although this was one of the few studies to focus on the depressive symptoms of patients with TB in China, our sample only comprised patients with TB in a central province. Therefore, it is necessary to conduct more representative studies to determine the status of depressive symptoms among patients with TB across China.

## Conclusion

In sum, we used SEM to explore the pathways linking family function, doctor-patient communication, and knowledge about TB to depressive symptoms and confirmed the mediating effect of stigma in the process. Awareness and management of depression in patients with TB may lead to better outcomes. There is an urgent need to develop effective interventions to prevent and decrease depression of patients. The current findings provide direction and information for depression interventions among patients with TB that improving family function and doctor-patient communication, increasing TB knowledge, and alleviating stigma may help to alleviate depressive symptoms among patients with TB. More research is needed to focus on psychological interventions for patients with TB and to assess the effectiveness of these interventions.

## Author contributions

YG and XY designed the study. LQ, QY, and YT participated in the acquisition of data. LQ conducted the data analysis and drafted the manuscript. ZL, YG, and XY revised the manuscript. All authors reviewed the manuscript.

### Conflict of interest statement

The authors declare that the research was conducted in the absence of any commercial or financial relationships that could be construed as a potential conflict of interest.
